# Supplementary material for: The Role of Systemic Microvascular Dysfunction in Heart Failure with Preserved Ejection Fraction
Source: Biomolecules. 2022 Feb 9;12(2):278. doi: 10.3390/biom12020278 (PMC8961612; doi:10.3390/biom12020278)
Supplement: Supplementary file 1 [file biomolecules-12-00278-s001.zip › biomolecules-1543024-SI.pdf]

biomolecules

Review

The role of systemic microvascular dysfunction in heart failure with preserved ejection fraction: Supplemental material

Jerremy Weerts, MD [1,*], Sanne G.J. Mourmans, MD [1], Arantxa Barandiarán Aizpurua, MD, PhD [1], Blanche L.M. Schroen, PhD [1], Christian Knackstedt, MD, PhD [1], Etto Eringa, PhD [2,3], Alfons J.H.M. Houben, PhD [4], Vanessa P.M. van Empel, MD, PhD [1]

1 Department of Cardiology, CARIM School for Cardiovascular Diseases, Maastricht University Medical Centre (MUMC+), Maastricht, the Netherlands.
2 Department of Physiology, CARIM School for Cardiovascular Diseases, Maastricht University, Maastricht, the Netherlands.
3 Department of Physiology, Amsterdam Cardiovascular Sciences, Amsterdam University Medical Center, Amsterdam, the Netherlands.
4 Department of Internal Medicine, CARIM School for Cardiovascular Diseases, Maastricht University Medical Centre (MUMC+), Maastricht, the Netherlands.
* Correspondence: jerremy.weerts@mumc.nl; Tel.: +31 43 387 7097

Contents

Supplemental Methods 2

Supplemental Table S1. Techniques and stimuli used in clinical research to assess microvascular function in HFpEF .. 3

Supplemental Table S2. Studies on peripheral microvascular function in HFpEF 4

Supplemental Table S3. Studies on coronary microvascular function in HFpEF 8

Supplemental Table S4. Intervention studies in HFpEF targeting the nitric oxide – protein kinase G pathway 13

References 17

Supplemental Methods

Literature search

The initial literature search for this narrative review was performed to capture all studies with results on cardiac and peripheral microvascular function in humans with heart failure with preserved ejection fraction (HFpEF) in PubMed and Google Scholar. In addition, a literature search was performed to find interventional clinical trials targeting aspects of the microvascular function in HFpEF. Finally, additional literature was searched according to microvascular aspects (in HFpEF). The different relevant searches are provided below.

Initial literature search:

((((((((hfpef) OR diastolic dysfunction) OR "Heart Failure, Diastolic"[Mesh]) OR diastolic heart failure) OR diastolic heart failures) OR heart failure preserved) OR heart failure preserved ejection fraction) OR heart failure with preserved ejection fraction)) AND (((((((((((endothelial) OR endothelial dysfunction) OR microvascular) OR coronary microvascular dysfunction) OR coronary microcirculation) OR peripheral microcirculation) OR microvascular obstruction) OR microvascular dysfunction) OR microcirculation) OR endothelium) OR endothelium dysfunction) OR nitric oxide)

Interventional clinical trials searches:

<u>Sodium glucose transport protein 2 inhibitors:</u> ((((Soluble guanylate cyclase stimulators) OR (sgc stimulator)) OR (vericiguat)) OR (praliciguat)) AND (((heart failure) OR (heart failure preserved ejection fraction)) OR (HFpEF)) Filters: Randomized Controlled Trial

((heart failure preserved ejection fraction) OR (HFpEF) AND (((((sglt-2 inhibitor) OR (sodium glucose transport protein 2 inhibition)) OR (empagliflozin)) OR (dapagliflozin)) OR (Canagliflozin))

<u>Nitrates:</u> (((((isosorbide dinitrate) OR (isosorbide mononitrate)) OR (inorganic nitrate)) OR (organic nitrate)) OR (inorganic nitrite)) AND (((heart failure) OR (heart failure preserved ejection fraction)) OR (HFpEF)) Filters: Randomized Controlled Trial

<u>Angiotensin receptor neprilysin inhibitors:</u> (((sacubitril valsartan) OR (arni)) OR (angiotensin receptor neprilysin inhibitor)) AND (((heart failure) OR (heart failure preserved ejection fraction)) OR (HFpEF)) Filters: Randomized Controlled Trial

<u>Phosphodiesterase inhibitors:</u> (((heart failure) OR (heart failure preserved ejection fraction)) OR (HFpEF)) AND (((sildenafil) OR (pde inhibitor)) OR (phosphodiesterase inhibitor)) Filters: Randomized Controlled Trial

Table S1. Techniques and stimuli used in clinical research to assess microvascular function in HFpEF.

	TISSUE	TECHNIQUE	STIMULUS	PARAMETER
PERIPHERAL	Skin	Laser Doppler flowmetry	Acetylcholine	Endothelium-dependent hyperemia [1]
			Sodium nitroprusside	Endothelium-independent hyperemia [1]
			Ischemia, heat	Index of cutaneous perfusion (reactive hyperemia) [2]
		EndoPAT	Ischemia	Reactive hyperemia [3-9]
	Thigh muscle	Histology	None	Anatomical capillary density, capillary-to-fibre ratio [10]
		Near-infrared spectroscopy	Muscle contraction	Haemoglobin O2 content [11]
	Retina	Fundoscopy	None, flicker light	Vessel diameter [12-15]
			None	Microbleeds [15]
CARDIAC	Myocardium	Coronary angiogram, echo Doppler, Magnetic resonance imaging	Adenosine	Coronary flow reserve (maximal hyperemia) [7, 16-23]
		Coronary angiogram	Adenosine	Index of microcirculatory resistance (minimal resistance) [16, 17, 20]
			Acetylcholine	Endothelium-dependent hyperemia [18, 19]
			None, nitroglycerin	Total myocardial blush grade score (myocardial perfusion) [24]
		Positron emission tomography	Adenosine, dipyridamole	Maximal hyperemia, active hyperemia [25, 26]
		Histology (post-mortem)	None	Microvascular density [27]

Table S2. Studies on peripheral microvascular function in HFpEF

Study design	Definition of HFpEF	HFpEF population	Control population	Method (measurement)	Stimulus	Protocol	Microvascular function assessed	Outcome (SD/IQR)	Outcome adjusted for confounders
				Skin-finger					
Prospective [4]	HFnEF ESC 2007 diagnostic criteria [28] and no presence of COPD	HFpEF (n=321)	Controls without HF, matched for age, sex, hypertension, and DM (n=173)	Peripheral arterial tonometry (endoPAT): (RHI)	Ischemia	Prior medication intake, fasting state.	Hyperemia	Log RHI: 0.53±0.20 vs. 0.64±0.20, p < 0.001	Age, sex, HT, DM
Prospective [7]	NYHA ≥2; LVEF ≥40%; and HF hospitalization (with structural heart disease on echo), or elevated BNP (various cut-offs, SR NT-proBNP ≥ 300ng/L), or elevated PCWP (rest/exercise), or E/e' ratio ≥ 15 (rest)	HFpEF (n=202)	No controls	endoPAT (RHI)	Ischemia	No details.	Hyperemia	Log RHI: no absolute values reported. Correlation with CFR of R 0.21, p = 0.004	
Retrospective [5]	Framingham HF criteria and HFnEF ESC 2007 diagnostic criteria, and history of HF hospitalization.	HFpEF (159)	No controls	endoPAT (RHI)	Ischemia	No eating or drinking 8 hours prior measurement, only water.	Hyperemia	Log RHI: 0.50±0.09 Event free 0.52±0.09 vs. Events 0.46±0.08, p < 0.001	

Prospective (cross-sectional) [6]	HFnEF ESC 2007 diagnostic criteria and history of HF hospitalization	HFpEF (n=62)	Controls matched for age, sex, HT, DM, dyslipidemia and CAD (n=64)	endoPAT (RHI)	Ischemia	No details.	Hyperemia	RHI: 2.01 [1.64-2.42] vs. 1.70 [1.55-1.88], p < 0.001	Age, sex, HT, DM, dislipidemia, CAD
Prospective [9]	LVEF >50%, Framingham HF criteria [29]	HFpEF (n=42)	HFrEF (n=46)	endoPAT (RHI)	Ischemia	Prior to cardiac catheterization, overnight fasting and dietary restrictions, standardized room and body position	Hyperemia	RHI: 1.77 [1.67-2.16] vs. 1.53 [1.42-1.94], p = 0.014.	
Prospective [8]	ESC 2012 diagnostic criteria [29, 30], with sedentary lifestyle.	HFpEF (n=26)	Healthy controls, matched for age and sex (n=26)	endoPAT (RHI)	Ischemia	Prior and after CPET, no other details.	Hyperemia	RHI interpretation from boxplots: 1.9 [1.6-2.9] vs. 1.8 [2.0-3.3], p = 0.036. No effect of exercise	Age and sex
Prospective [3]	LVEF >50%, Framingham HF criteria	HFpEF (n=21)	HT controls without HF (n=19) Healthy controls (n=10)	endoPAT (RHI)	Ischemia	Cardiovascular medicine withheld 24h prior, fasting state.	Hyperemia	Log RHI: 0.85±0.42 vs. 0.92±0.38 vs. 1.33±0.34, p = n.s. between HFpEF and HT controls	History of CAD, peak VO2.
Skin-arm									

Prospective [2]	Framingham HF criteria and prior HF hospitalization, excluding AF.	HFpEF (n=45)	HT controls, matched for age, sex and diabetic status (n=45)	Laser Doppler flowmetry (LDF), power spectral density (PSD) of the LDF signal	None, ischemia	6-8 weeks after HF hospitalization, room temperature maintained at 22 degrees Celsius.	Vasomotion, hyperemia	LDF PSD: lower in HFpEF, no absolute numbers reported, p < 0.05. Peak blood flow (PU): 135 [104-206] vs. 177 [139-216], p = 0.03	Beta-blocker, loop diuretics, CAD
Prospective [1]	Signs or symptoms of HF by Boston criteria, diuretic therapy, increased NTproBNP, and LVEF ≥50%. All had coronary artery disease by study design.	HFpEF with CAD (n=12)	HFrEF with CAD (n=12) CAD without HF (n=12)	Laser Doppler imaging (LDI) coupled with transcutaneous iontophoresis of vasodilators	acetylcholine, sodium nitroprusside	No morning medication prior and no caffeine and tobacco 12 hours prior assessment. Controlled temperature (21-23 degrees Celsius) after 30 min rest.	Hyperemia	Vasodilation due to Acth: No absolute values reported. P = 0.00099 (HF vs controls). Vasodilation due to nitroprusside: p = 0.006 (HF vs controls)	
				Muscle-leg					

Prospective [10]	Signs and symptoms of HF according to the National Health and Nutrition Examination Survey HF clinical score of ≥3, LVEF ≥50%, no other etiology mimicking HF symptoms	HFpEF (n=22)	Healthy controls: age-matched, no chronic medical illness, chronic medication, complaints and abnormal findings during screening tests, or regularly undertook vigorous exercise (n=43).	Histology (skeletal muscle biopsy of thigh)	Biopsy after an overnight fast, no medication affecting bleeding time.	Capillary density	Capillary-to-fibre ratio: 1.35±0.32 vs. 2.53±1.37, p = 0.006
Prospective [11]	LVEF >50%, excluding pacemakers and respiratory disease.	HFpEF (n=7)	No controls.	Near-infrared spectroscopy: index for skeletal muscle hemoglobin oxygenation of thigh	Consumption of a light meal, and no caffeine 12 hours prior assessments	Diffusion	Muscle deoxygenation overshoot was decreased after priming exercise, p = 0.041

Abbreviations: BNP, B-type natriuretic peptide; CAD, coronary artery disease; CFR; coronary flow reserve; COPD, chronic obstructive pulmonary disease; CPET, cardio-pulmonary exercise test; DM, diabetes mellitus; ESC, European Society of Cardiology; FMD, flow-mediated vasodilation; HF, heart failure; HFpEF, heart failure with preserved ejection fraction; HFrEF, heart failure with reduced ejection fraction; HT, hypertension; LV, left ventricle/ventricular; LVEF, left ventricular ejection fraction; MVD, microvascular disease; NT-proBNP, N-terminal-pro hormone BNP; NYHA, New York Heart Association class; PCWP, pulmonary capillary wedge pressure; RHI, Reactive hyperemia index; SR, sinus rhythm; VO2, oxygen uptake.

Table S3. Studies on coronary microvascular function in HFpEF.

Study design	Definition of HFpEF	Study population	Method (measurement)	Stimulus	Microvascular function assessed	Outcome (SD/IQR)	Outcome adjusted for confounders	Prevalence of MVD in HFpEF
Autopsy								
Retrospective [27]	Previous HF hospitalization OR outpatient diagnosis of HF; and LVEF ≥40%	Deceased: HFpEF (n=124) Controls (no HF) (n=104)	ECAM-1 bright-field vessel detection: microvessels/mm2 (microvascular density)		Rarefaction	Microvascular density: 961 (800-1370) vs. 1316 (1148-1467), p <0.0001	Not performed, unmatched population	Not reported
Invasive coronary assessments								
Retrospective [17]	Signs and symptoms of HF, LVEF > 50%, LVEDI <97mL/m2, LVEDP > 16mmhg (LHC).	CAG after positive stress test: HFpEF >65 (n=32) HFpEF <65 (n=24) Controls (n=31)	Invasive CFR and IMR	Adenosine	Hyperemia	CFR: 1.94±0.28 vs. 1.83±0.32 vs. 3.24±1.11, p ≤ 0.04<br><br>IMR: 39.2±6.8 vs. 27.2±6.4 vs. 18.3±4.4, p ≤ 0.03	Age, sex, HT, DM, CKD, AF, BMI, LVMI. Unmatched controls	Not reported.
Retrospective [18]	Symptoms of HF, LVEF ≥50%, at least one of the following: PCWP ≥ 15, LVEDP ≥18. NTproBNP >400pg/ml or BNP > 200pg/ml, E/e′ ≥15, LAVI >34.	HFpEF (n=162)	Invasive CFR and coronary blood flow (CBF)	Adenosine, acetylcholine	Hyperemia	No absolute values reported. Mortality is increased in coronary MVD (HR 2.8-3.5).	Age, sex, BMI, DM, HT, hyperlipidemia, smoking, Hb, creatinin, uric acid	72% (CBF <0% increase and/or CFR <2.5)
Retrospective [31]	Unexplained cardiac exertion symtpoms, LVEF > 50%, pulmonary arterial wedge pressure ≥ 15 mmHg at rest / ≥ 25	Indication for coronary reactivity testing and invasive haemodynamic evaluation during	Invasive CFR and CBF	Adenosine, acetylcholine	Hyperemia	CFR: 2.5±0.6 vs. 3.2±0.7, p = 0.0003 Median CBF % increase: 1 (-	Age, sex	46% endothelial independent CMD (CFR < 2.5), 86% endothelial dependent CMD

	mmHg at peak exercise.	exercise. HFpEF (n=22), no HFpEF (n=29)				35;34) vs. 64 (-4;133), p = 0.002		(CBF < 50% increase)
Prospective [32]	Hospitalized HFpEF, ESC 2016 diagnostic criteria [33].	HFpEF with obstructive epicardial CAD (n=38), HFpEF without epicardial CAD (n=37)	CAG (CFR, coronary reactivity, IMR) and MRI	Adenosine, acetylcholine	Hyperemia	CFR: 2.0(1.2-2.4) vs. 2.4(1.5-3.1),p = 0.06. IMR: 18(12-26) vs. 27(19-43), p = 0.02. 24% microvascular spasm due to Acth.	Clinical characteristics are compared between groups based on coronary results.	91% had evidence of epicardial CAD, CMD, or both. 85% had any CMD. Endothelium-dependent CMD (20-90% luminal constriction): 0% vs. 28%, p = 0.18 CFR <2.0 and/or IMR ≥25: 62% vs 69%, p = 0.52
Prospective (cross-sectional) [16]	LVEF≥50, Framingham criteria for HF, BNP >100pg/ml or previous intravenous diuretics use	Clinical indication for CAG: HFpEF (n=30) Controls (n=14)	Invasive CFR and IMR	Adenosine	Hyperemia	CFR: 2.55±1.60 vs. 3.84±1.89, p = 0.024 IMR: 26.7±10.3 vs. 19.7±9.7, p = 0.037	Explanatory analysis on age, BMI, GFR, BNP, echocardiographic data, hemodynamic data. Unmatched controls	73% (CFR ≤2.0 and/or IMR ≥23)
Retrospective [24]	ESC 2012 and AHA 2013 diagnostic criteria: Signs and symptoms of HF, LVEF >50%, LVEDI <97mL/m2, evidence of reduced diastolic LV function (mitral inflow pattern, tissue velocities, E/e	Patients with angina presented to the emergency unit: HFpEF (n=155) Controls (n=135)	Total myocardial blush grade score (TMBGS)	None, nitroglycerin	Blood flow	TMBGS: 5.6±1.22 vs. 6.1±1.26, p = 0.02	Not performed, unmatched population	Not reported

	ratio, LAVI, LV mass index)							
**Non-invasive coronary assessments**								
Prospective [34]	Framingham criteria for HF, NYHA 2-3b, LVEF ≥50%	HFpEF (n=19) Matched healthy controls (n=19)	PET (C-acetate-11): myocardial blood flow (MBF) and myocardial oxygen consumption (MVO_2)	Dobutamine	Blood flow, hyperemia, diffusion	MBF increase: 78% vs. 151%, p = 0.0480 MVO2 increase: 59% vs. 86%, p = 0.0079 Absolute values during stress test not significantly different.	LVH, Hb. Healthy controls were matched for age and sex.	Not reported
Retrospective [25]	LVEF ≥ 50%, NYHA ≥1, confirmed HFpEF diagnosis based on medical records. Significant valve disease not excluded.	Indication for cardiac PET: HFpEF (n=78) HT without HF (n=112) No HF no HT (n=186)	PET (Rb-82): global myocardial flow reserve (MFR)	Dipyridamole	Hyperemia	MFR: 2.16±0.69 vs. 2.54±0.80 vs. 2.89±0.70, p ≤ 0.001	Age, sex, BMI, smoking, DM, HT, hyperlipidaemia, HT, AF, statin use. Controls matched for HT.	40% (MFR <2.0)
Retrospective [26]	LVEF >50% and HF hospitalization	Suspected CAD: Cohort without HF (n=201)	PET (Rb-82): (CFR)	Regadenoson or dipyridamole	Hyperemia	18% of the patients had a HFpEF event during follow-up. Independent HR with CFR < 2.0 of 2.47 (1.09-5.62)	In entire cohort: AF, CKD, troponin, LVEF, CFR, E/e' septal	Not reported
Prospective [21]	ESC 2007 diagnostic criteria: LVEF >50% and E/e'≥15 or 8<E/e'<15 and BNP >200pg/dL.	HFpEF (n=25) LVH (n=13) Controls (n=18)	MRI (CFR)	Adenosine	Hyperemia	CFR: 2.21±0.55 vs. 3.05±0.74 vs. 3.83±0.73, p ≤ 0.002	BNP, LVEF, E/e', LA dimension	76% (CFR <2.5)

Retrospective [35]	ESC 2016 diagnostic criteria	HFpEF without events (n=137) with events (n=26)	MRI (CFR)	Adenosine	Hyperemia	CFR: 2.67±0.64 vs. 1.93±0.38	Not performed	3% vs. 42% (CFR <2.0)
Prospective [36]	LVEF >40% and presence of LVH and/or extracellular volume fraction > 28% on T1-mapping; dyspnea; history of HT and ≥1 additional cardiovascular risk factor	HFpEF (n=6) Post MI (n=6) Healthy controls (n=10 Gadofosveset, n=10 Gadobutrol))	MRI: intravascular volume of basal septum (IVV)	Gadofosveset	Permeability	IVV: 0.155±0.033 vs. 0.146±0.038 vs. 0.135±0.018, p = 0.413	Not performed, unmatched controls	Not reported
Prospective [7]	NYHA ≥2; LVEF ≥40%; and HF hospitalization (with structural heart disease on echo), or elevated BNP (various cut-offs, SR NTproBNP ≥ 300ng/L), or elevated PCWP (rest/exercise), or E/e' ratio ≥ 15 (rest)	HFpEF (n=202)	Echocardiography (CFR)	Adenosine	Hyperemia	CFR: 2.13±0.51	Age, sex, BMI, AF, DM, CAD, smoking, LV mass, 6MWT, KCCQ, urinary albumin-creatinin ratio. No controls.	75% (CFR <2.5)
Prospective [22]	ESC 2012 diagnostic criteria	HFpEF (n=77) Healthy controls (n=30)	Echocardiography (CFR)	Adenosine	Hyperemia	CFR: 1.7±0.2 (with MVD) vs. 3.1±0.4 (no MVD) vs. 3.4±0.3 (control)	Age, LAVI, LVMI, LVEF, E/e', 6MWT distance	66% (CFR ≤2.0)

Abbreviations: AF, atrial fibrillation; AHA, American Heart Association; BMI, body mass index; BNP, B-type natriuretic peptide; CAD, coronary artery disease; CAG, coronary angiography; CFR; coronary flow reserve; CKD, chronic kidney disease; CMD, coronary microvascular dysfunction; DM, diabetes mellitus; ECAM, endothelial cell adhesion molecule; ESC, European Society of Cardiology; GFR, glomerular filtration rate; HF, heart failure; HFpEF, heart failure with preserved ejection fraction; HR, hazard ratio; HT, hypertension; IMR, index of microcirculatory resistance; LAVI; left atrial volume index; LHC, left-sided heart catheterisation; LV, left ventricle/ventricular;

LVEDI, left ventricular end-diastolic volume index; LVEDP, Left ventricular end-diastolic pressure; LVEF, left ventricular ejection fraction; LVMI, left ventricular mass index; MFR, myocardial flow reserve; MVD, microvascular disease; NT-proBNP, N-terminal-pro hormone BNP; NYHA, New York Heart Association class; PCWP, pulmonary capillary wedge pressure; PET, positron emission tomography; SR, sinus rhythm.

Table S4. Intervention studies in HFpEF targeting the nitric oxide – protein kinase G pathway

Study	Definition of HFpEF	Study size	Study design	Drug	Outcomes	Result (time)
Soluble guanylate cyclase (sGC) stimulators						
SOCRATES-PRESERVED [37]	LVEF ≥ 45%, NYHA II-IV, elevated NT-pro BNP/ BNP, within 4 weeks of HF hospitalisation/ IV diuretic treatment for worsening HF	n = 477	Multinational phase 2b randomized, double-blind, placebo controlled, dose-finding trial	Vericiguat (1.25mg, 2.5mg, 5mg, 10mg) vs. placebo	NT-proBNP, LAV<br>KCCQ	Neutral (12wks)<br>Positive (12wks)
VITALITY-HFpEF [38]	LVEF ≥ 45%, NYHA class II-III symptoms, within 6 months of recent decompensation, elevated natriuretic peptides	n = 789	Multicentre phase 2b randomized, double-blind, placebo controlled trial	Vericiguat (10mg, 15mg) vs. placebo	PLS, KCCQ	Neutral (24wks)
CAPACITY-HFpEF [39]	LVEF ≥ 40%, NYHA II-IV AND 1) HF hospitalisation (12months) or 2) elevated NT-proBNP / BNP, AND 1) LVH or 2) LA enlargement, 3) diastolic dysfunction on cardiac ultrasound, 4) elevated PCWP at rest/exercise, AND peak VO2 < 80% 2)RER ≥ 1 determined by CPET	n = 196	Phase 2, randomized, double-blind, placebo-controlled trial	Praliciguat (40mg) vs. placebo	Peak VO2, 6MWD, functional and structural echocardiographic endpoints, KCCQ	Neutral (12wks)
Sodium-glucose transport protein 2 (SGLT-2) inhibitors						
MUSCAT-HF [40]	LVEF ≥ 45%, BNP ≥ 35 pg/ml. (only inclusion of patients with DM type II)	n = 173	open-label, multicentre, randomized controlled trial	Luseogliflozin (2.5mg) vs.voglibose (0.6mg)	Difference from baseline in BNP level	Neutral (12wks)
CANONICAL study [41]	LVEF ≥ 50%, history of heart failure, E/e'average > 14 or lateral e'< 10cm/s, elevated BNP / NT proBNP and NYHA II-III within 8 weeks before consent (only inclusion of patients with DM type II)	n = 82	Multicentre, randomized, open label, parallel-group controlled trial	Canaglifozin (100mg) vs standard therapy	Difference in body weight<br>BNP levels	Positive (24wks) (↓body weight)<br>Neutral (24wks)

PRESERVED-HF [42]	LVEF ≥ 45%, NYHA II-IV, elevated (NT-pro)BNP, required diuretic therapy, HF hospitalisation/ urgent HF visit with IV diuretic treatment <12 months, and elevated filling pressures on RHC or echocardiographic structural heart abnormalities.	n = 324	Multicentre randomized, double-blind, placebo-controlled trial	Dapagliflozin (10mg) vs. Placebo	KCCQ clinical and overall summary score, 6MWD	Positive (12wks)
EMPERIAL-PRESERVED [43]	LVEF > 40%, NYHA II-IV, HF diagnosed ≥3 months before screening, 6MWD ≥ 100m and ≤350m at screening	n = 315	Phase 3, randomized, double-blind, placebo-controlled trial	Empagliflozin (10mg) vs. Placebo	6MWD, KCCQ total symptom score, CHFQ dyspnoea score	Neutral (12 wks)
EMPEROR-PRESERVED [44] [45]	LVEF ≥ 40%, NYHA II-IV, chronic HF for at least 3 months, elevated NT-proBNP, and evidence of structural heart changes on echocardiography (increased LA size or LV mass) or HF hospitalisation within 12 months of screening	n = 5942 / 5988	Phase 3 international, multicentre, randomized, double-blind, parallel-group, placebo-controlled trial	Empagliflozine (10mg) vs. Placebo	KCCQ<br>combined cardiovascular death or<br>HF hospitalisation	Positive (52wks)<br>Positive (mean 26 months)
Sacubitril (angiotensin receptor neprilysin inhibitor, ARNI)-valsartan						
PARAMOUNT [46]	LVEF ≥ 45%, NYHA II-III, NT pro BNP > 400 pg/mL	n = 301	Phase 2, randomized, parallel-group, double-blind multicentre trial	Sacubitril-valsartan (400mg) vs valsartan (320 mg)	NT-proBNP	Positive (36wks)
PARAGON-HF [47]	LVEF ≥ 45%, NYHA II-IV, elevated natriuretic peptides and structural heart disease	n = 4822	Randomized, double-blind, active-comparator trial	sacubitril-valsartan (400mg) vs valsartan (320) mg	Composite HF hospitalisations and death from cardiovascular cause	Neutral (35 months)
PARALLAX [48]	LVEF ≥ 40%, NYHA II-IV elevated NT-proBNP, structural heart disease (LA enlargement or LV hypertrophy on echocardiography), reduced quality of life	n = 2572	Randomized, double-blind, parallel group clinical trial	sacubitril-valsartan (400mg) vs angiotensin receptor blocker or placebo	NT-proBNP<br>6MWD, quality of life	Positive (24wks)<br>Neutral (24wks)
Phosphodiesterase (PDE) inhibitors						

RELAX trial [49] [50]	LVEF ≥ 50%, stable outpatient, elevated NT-proBNP or invasively measured filling pressures, and reduced exercise capacity (peak VO2 ≤ 60% of predicted)	n = 216 / 48	Multicentre, double-blind, placebo-controlled, parallel-group, randomized clinical trial	Sildenafil (PDE5-inhibitor) (20mg, 60 mg) vs. Placebo	peak oxygen consumption, 6MWD, Clinical status rank score.<br>Ventricular- vascular structure/function (n=48)<br>Endothelial function, arterial tonometry, exercise capacity (n=48)	Neutral (24wks)<br>↓arterial elastance<br>Other analyses/: neutral
MilHFPEF [51]	LVEF ≥ 50%, NYHA III, echocardiographic requirements according to ESC 2016, HF hospitalisation < 12 months or elevated natriuretic peptides, on stable HF therapy for 2 weeks before screening	n = 23	randomized, double-blind, placebo-controlled trial	Milrinone (PDE3-inhibitor) (14mg) vs placebo	KCCQ summary score<br>6MWD	Positive (4wks)<br>Neutral (4wks)
			Nitrates			
NEAT- HFpEF [52]	LVEF ≥ 50%, stable medical therapy, and 1 of the following within 12 months of enrolment: HF hospitalisation with radiographic evidence of pulmonary congestion, elevated LV end diastolic pressure or pulmonary capillary wedge pressure, elevated natriuretic peptides or echocardiographic evidence of diastolic dysfunction	n = 110	Multicentre, double-blind, crossover study	(organic) isosorbide mononitrate (30mg, 60mg, 120mg) vs. placebo	Daily activity level (accelerometer)<br>Quality of life, 6MWD, NT-proBNP	↓daily activity level (6wks)<br>Other endpoints: Neutral (6wks)
Zamani et al. [53]	LVEF ≥ 50%, stable medical therapy for 1 month, and 1 of the following: prior HF hospitalisation, acute HF treatment with IV diuretics or hemofiltration, echocardiographic evidence of elevated filling pressures, chronic use of loop diuretics, or elevated NT-proBNP	n = 44	Randomized, double-blind pilot clinical trial	(organic) isosorbide dinitrate (120mg), isosorbide dinitrate + hydralazine (direct-acting smooth muscle relaxant) (120mg + 75mg) or placebo	Change in reflection magnitude (arterial tonometry / Doppler echocardiography), LV mass and fibrosis, KCCQ 6MWD	Neutral (6mo)<br>Negative in isosorbide dinitrate + hydralazin (other arms: neutral) (6mo)

Zamani et al. [54]	LVEF ≥ 50%, symptoms of heart failure, E/e'> 8 on echocardiography, and 1 of the following: LAVI > 34 mL/m2, elevated NT proBNP (<1yr), long term use of loop diuretics or elevated filling pressures on cardiac catheterization	n = 17	Randomized, double-blind, crossover study	Inorganic NO3- in beetroot juice (12.9mmol) vs. placebo	Peak VO2, Total work performed<br>Exercise efficiency	Positive (3hr)<br>Neutral (3hr)
Borlaug et al. [55]	LVEF ≥ 50%, symptoms of heart failure, increased PCWP at rest (>15 mmHg) and/or with exercise (≥ 25 mmHg)	n = 28	Randomized, double-blind, placebo-controlled, parallel-group trial	IV sodium inorganic nitrite (µg/kg/min for 5 min) vs. placebo	pulmonary capillary wedge pressure during exercise	Positive (10min)
INDIE-HFpEF [56]	LVEF ≥ 50%, stable medical therapy, chronic use of loop diuretic and 1 of the following: elevated natriuretic peptides, elevated LV end diastolic pressure or pulmonary capillary wedge pressure, previous HF hospitalisation or radiographic evidence of pulmonary congestion, or echocardiographic diastolic dysfunction	n = 105	Multicentre, double-blind, placebo-controlled, 2-treatment, crossover trial	inhaled nebulized inorganic nitrite (138mg and 240 mg) vs. placebo	peak VO2, daily activity levels (accelerometry), KCCQ, Cardiac filling pressures (echo), NT-proBNP	Neutral (4wks)

Abbreviations: 6-MWD, 6-minute walking distance; BNP, B-type natriuretic peptide; CHFQ, Chronic Heart Failure Questionnaire Self-Administered Standardized format; CPET, cardiopulmonary exercise test; DM, diabetes mellitus; HF, heart failure; IV, intravenous; KCCQ, Kansas City Cardiomyopathy Questionnaire; LA, left atrial/atrium; LAV, left atrial volume; LV, left ventricle/ventricular; LVEF, left ventricular ejection fraction; LVH, left ventricular hypertrophy; NT-proBNP, N-terminal prohormone of brain natriuretic peptide; NYHA class, New York heart association class; PCWP, pulmonary capillary wedge pressure; PSL, physical limitation score; RER, respiratory exchange ratio; RHC, right heart catheterisation; VO2, oxygen consumption.

References

1. Balmain, S., et al., *Differences in arterial compliance, microvascular function and venous capacitance between patients with heart failure and either preserved or reduced left ventricular systolic function.* Eur J Heart Fail, 2007. **9**(9): p. 865-71.
2. Marechaux, S., et al., *Vascular and Microvascular Endothelial Function in Heart Failure With Preserved Ejection Fraction.* J Card Fail, 2016. **22**(1): p. 3-11.
3. Borlaug, B.A., et al., *Global cardiovascular reserve dysfunction in heart failure with preserved ejection fraction.* J Am Coll Cardiol, 2010. **56**(11): p. 845-54.
4. Akiyama, E., et al., *Incremental prognostic significance of peripheral endothelial dysfunction in patients with heart failure with normal left ventricular ejection fraction.* J Am Coll Cardiol, 2012. **60**(18): p. 1778-86.
5. Matsue, Y., et al., *Endothelial dysfunction measured by peripheral arterial tonometry predicts prognosis in patients with heart failure with preserved ejection fraction.* Int J Cardiol, 2013. **168**(1): p. 36-40.
6. Yamamoto, E., et al., *The pivotal role of eNOS uncoupling in vascular endothelial dysfunction in patients with heart failure with preserved ejection fraction.* Int J Cardiol, 2015. **190**: p. 335-7.
7. Shah, S.J., et al., *Prevalence and correlates of coronary microvascular dysfunction in heart failure with preserved ejection fraction: PROMIS-HFpEF.* Eur Heart J, 2018. **39**(37): p. 3439-3450.
8. Gevaert, A.B., et al., *Endothelial dysfunction and cellular repair in heart failure with preserved ejection fraction: response to a single maximal exercise bout.* Eur J Heart Fail, 2019. **21**(1): p. 125-127.
9. Waku, R., et al., *Flow-Mediated Vasodilation and Reactive Hyperemia Index in Heart Failure with Reduced or Preserved Ejection Fraction.* Tohoku J Exp Med, 2020. **252**(1): p. 85-93.
10. Kitzman, D.W., et al., *Skeletal muscle abnormalities and exercise intolerance in older patients with heart failure and preserved ejection fraction.* Am J Physiol Heart Circ Physiol, 2014. **306**(9): p. H1364-70.
11. Boyes, N.G., et al., *Effects of heavy-intensity priming exercise on pulmonary oxygen uptake kinetics and muscle oxygenation in heart failure with preserved ejection fraction.* Am J Physiol Regul Integr Comp Physiol, 2019. **316**(3): p. R199-R209.
12. Nagele, M.P., et al., *Retinal microvascular dysfunction in heart failure.* Eur Heart J, 2018. **39**(1): p. 47-56.
13. Cheung, N., et al., *Retinal Arteriolar Narrowing and Left Ventricular Remodeling: The Multi-Ethnic Study of Atherosclerosis.* Journal of the American College of Cardiology, 2007. **50**(1): p. 48-55.
14. Chandra, A., et al., *The association of retinal vessel calibres with heart failure and long-term alterations in cardiac structure and function: the Atherosclerosis Risk in Communities (ARIC) Study.* Eur J Heart Fail, 2019. **21**(10): p. 1207-1215.
15. Wong, T.Y., et al., *Retinopathy and risk of congestive heart failure.* JAMA, 2005. **293**(1): p. 63-9.
16. Dryer, K., et al., *Coronary microvascular dysfunction in patients with heart failure with preserved ejection fraction.* Am J Physiol Heart Circ Physiol, 2018. **314**(5): p. H1033-H1042.
17. Xu, Z., et al., *Increased index of microcirculatory resistance in older patients with heart failure with preserved ejection fraction.* J Geriatr Cardiol, 2018. **15**(11): p. 687-694.
18. Yang, J.H., et al., *Endothelium-dependent and independent coronary microvascular dysfunction in patients with heart failure with preserved ejection fraction.* Eur J Heart Fail, 2020. **22**(3): p. 432-441.
19. Ahmad, A., et al., *Coronary microvascular dysfunction is associated with exertional haemodynamic abnormalities in patients with heart failure with preserved ejection fraction.* Eur J Heart Fail, 2021. **23**(5): p. 765-772.
20. Rush, C.J., et al., *Prevalence of Coronary Artery Disease and Coronary Microvascular Dysfunction in Patients With Heart Failure With Preserved Ejection Fraction.* JAMA Cardiology, 2021. **6**(10): p. 1130-1143.
21. Kato, S., et al., *Impairment of Coronary Flow Reserve Evaluated by Phase Contrast Cine-Magnetic Resonance Imaging in Patients With Heart Failure With Preserved Ejection Fraction.* J Am Heart Assoc, 2016. **5**(2).
22. Mahfouz, R.A., M. Gouda, and M. Abdelhamid, *Relation of microvascular dysfunction and exercise tolerance in patients with heart failure with preserved ejection fraction.* Echocardiography, 2020. **37**(8): p. 1192-1198.
23. Kato, S., et al., *Cardiovascular magnetic resonance assessment of coronary flow reserve improves risk stratification in heart failure with preserved ejection fraction.* Journal of Cardiovascular Magnetic Resonance, 2021. **23**(1): p. 112.
24. Sucato, V., et al., *Angiographic Evaluation of Coronary Microvascular Dysfunction in Patients with Heart Failure and Preserved Ejection Fraction.* Microcirculation, 2015. **22**(7): p. 528-33.
25. Srivaratharajah, K., et al., *Reduced Myocardial Flow in Heart Failure Patients With Preserved Ejection Fraction.* Circ Heart Fail, 2016. **9**(7).
26. Taqueti, V.R., et al., *Coronary microvascular dysfunction and future risk of heart failure with preserved ejection fraction.* Eur Heart J, 2018. **39**(10): p. 840-849.
27. Mohammed, S.F., et al., *Coronary microvascular rarefaction and myocardial fibrosis in heart failure with preserved ejection fraction.* Circulation, 2015. **131**(6): p. 550-9.
28. Paulus, W.J., et al., *How to diagnose diastolic heart failure: a consensus statement on the diagnosis of heart failure with normal left ventricular ejection fraction by the Heart Failure and Echocardiography Associations of the European Society of Cardiology.* Eur Heart J, 2007. **28**(20): p. 2539-50.
29. McKee, P.A., et al., *The Natural History of Congestive Heart Failure: The Framingham Study.* New England Journal of Medicine, 1971. **285**(26): p. 1441-1446.

30. Authors/Task Force, M., et al., *ESC Guidelines for the diagnosis and treatment of acute and chronic heart failure 2012: The Task Force for the Diagnosis and Treatment of Acute and Chronic Heart Failure 2012 of the European Society of Cardiology. Developed in collaboration with the Heart Failure Association (HFA) of the ESC.* European Heart Journal, 2012. **33**(14): p. 1787-1847.
31. Ahmad, A., et al., *Coronary microvascular dysfunction is associated with exertional haemodynamic abnormalities in patients with heart failure with preserved ejection fraction.* European Journal of Heart Failure, 2021. **23**(5): p. 765-772.
32. Rush, C.J., et al., *Prevalence of Coronary Artery Disease and Coronary Microvascular Dysfunction in Patients With Heart Failure With Preserved Ejection Fraction.* JAMA Cardiol, 2021. **6**(10): p. 1130-1143.
33. Ponikowski, P., et al., *2016 ESC Guidelines for the diagnosis and treatment of acute and chronic heart failure: The Task Force for the diagnosis and treatment of acute and chronic heart failure of the European Society of Cardiology (ESC). Developed with the special contribution of the Heart Failure Association (HFA) of the ESC.* Eur J Heart Fail, 2016. **18**(8): p. 891-975.
34. AbouEzzeddine, O.F., et al., *Myocardial Energetics in Heart Failure With Preserved Ejection Fraction.* Circ Heart Fail, 2019. **12**(10): p. e006240.
35. Kato, S., et al., *Cardiovascular magnetic resonance assessment of coronary flow reserve improves risk stratification in heart failure with preserved ejection fraction.* J Cardiovasc Magn Reson, 2021. **23**(1): p. 112.
36. Masci, P.G., et al., *Probing the intravascular and interstitial compartments of remodeled myocardium in heart failure patients with preserved and reduced ejection fraction: a CMR study.* BMC Med Imaging, 2019. **19**(1): p. 1.
37. Pieske, B., et al., *Vericiguat in patients with worsening chronic heart failure and preserved ejection fraction: results of the SOluble guanylate Cyclase stimulatoR in heArT failurE patientS with PRESERVED EF (SOCRATES-PRESERVED) study.* Eur Heart J, 2017. **38**(15): p. 1119-1127.
38. Armstrong, P.W., et al., *Effect of Vericiguat vs Placebo on Quality of Life in Patients With Heart Failure and Preserved Ejection Fraction: The VITALITY-HFpEF Randomized Clinical Trial.* Jama, 2020. **324**(15): p. 1512-1521.
39. Udelson, J.E., et al., *Effect of Praliciguat on Peak Rate of Oxygen Consumption in Patients With Heart Failure With Preserved Ejection Fraction: The CAPACITY HFpEF Randomized Clinical Trial.* Jama, 2020. **324**(15): p. 1522-1531.
40. Ejiri, K., et al., *Effect of Luseogliflozin on Heart Failure With Preserved Ejection Fraction in Patients With Diabetes Mellitus.* J Am Heart Assoc, 2020. **9**(16): p. e015103.
41. Ueda, T., et al., *Effect of the Sodium-Glucose Cotransporter 2 Inhibitor Canagliflozin for Heart Failure With Preserved Ejection Fraction in Patients With Type 2 Diabetes.* Circ Rep, 2021. **3**(8): p. 440-448.
42. Nassif, M.E., et al., *The SGLT2 inhibitor dapagliflozin in heart failure with preserved ejection fraction: a multicenter randomized trial.* Nat Med, 2021. **27**(11): p. 1954-1960.
43. Abraham, W.T., et al., *Effect of empagliflozin on exercise ability and symptoms in heart failure patients with reduced and preserved ejection fraction, with and without type 2 diabetes.* Eur Heart J, 2021. **42**(6): p. 700-710.
44. Butler, J., et al., *Empagliflozin, Health Status, and Quality of Life in Patients with Heart Failure and Preserved Ejection Fraction: The EMPEROR-Preserved Trial.* Circulation, 2021.
45. Anker, S.D., et al., *Empagliflozin in Heart Failure with a Preserved Ejection Fraction.* New England Journal of Medicine, 2021. **385**(16): p. 1451-1461.
46. Solomon, S.D., et al., *The angiotensin receptor neprilysin inhibitor LCZ696 in heart failure with preserved ejection fraction: a phase 2 double-blind randomised controlled trial.* Lancet, 2012. **380**(9851): p. 1387-95.
47. Solomon, S.D., et al., *Angiotensin-Neprilysin Inhibition in Heart Failure with Preserved Ejection Fraction.* N Engl J Med, 2019. **381**(17): p. 1609-1620.
48. Pieske, B., et al., *Effect of Sacubitril/Valsartan vs Standard Medical Therapies on Plasma NT-proBNP Concentration and Submaximal Exercise Capacity in Patients With Heart Failure and Preserved Ejection Fraction: The PARALLAX Randomized Clinical Trial.* JAMA, 2021. **326**(19): p. 1919-1929.
49. Redfield, M.M., et al., *Effect of phosphodiesterase-5 inhibition on exercise capacity and clinical status in heart failure with preserved ejection fraction: a randomized clinical trial.* JAMA, 2013. **309**(12): p. 1268-77.
50. Borlaug, B.A., et al., *Effects of sildenafil on ventricular and vascular function in heart failure with preserved ejection fraction.* Circ Heart Fail, 2015. **8**(3): p. 533-41.
51. Nanayakkara, S., et al., *Extended-Release Oral Milrinone for the Treatment of Heart Failure With Preserved Ejection Fraction.* J Am Heart Assoc, 2020. **9**(13): p. e015026.
52. Redfield, M.M., et al., *Isosorbide Mononitrate in Heart Failure with Preserved Ejection Fraction.* New England Journal of Medicine, 2015. **373**(24): p. 2314-2324.
53. Zamani, P., et al., *Isosorbide Dinitrate, With or Without Hydralazine, Does Not Reduce Wave Reflections, Left Ventricular Hypertrophy, or Myocardial Fibrosis in Patients With Heart Failure With Preserved Ejection Fraction.* J Am Heart Assoc, 2017. **6**(2).
54. Zamani, P., et al., *Effect of inorganic nitrate on exercise capacity in heart failure with preserved ejection fraction.* Circulation, 2015. **131**(4): p. 371-80; discussion 380.
55. Borlaug, B.A., K.E. Koepp, and V. Melenovsky, *Sodium Nitrite Improves Exercise Hemodynamics and Ventricular Performance in Heart Failure With Preserved Ejection Fraction.* J Am Coll Cardiol, 2015. **66**(15): p. 1672-82.
56. Borlaug, B.A., et al., *Effect of Inorganic Nitrite vs Placebo on Exercise Capacity Among Patients With Heart Failure With Preserved Ejection Fraction: The INDIE-HFpEF Randomized Clinical Trial.* Jama, 2018. **320**(17): p. 1764-1773.
